# Supplementary figures and images for: Potential and action mechanism of favipiravir as an antiviral against Junin virus
Source: PLoS Pathog. 2022 Jul 11;18(7):e1010689. doi: 10.1371/journal.ppat.1010689 (PMC9302769; doi:10.1371/journal.ppat.1010689)

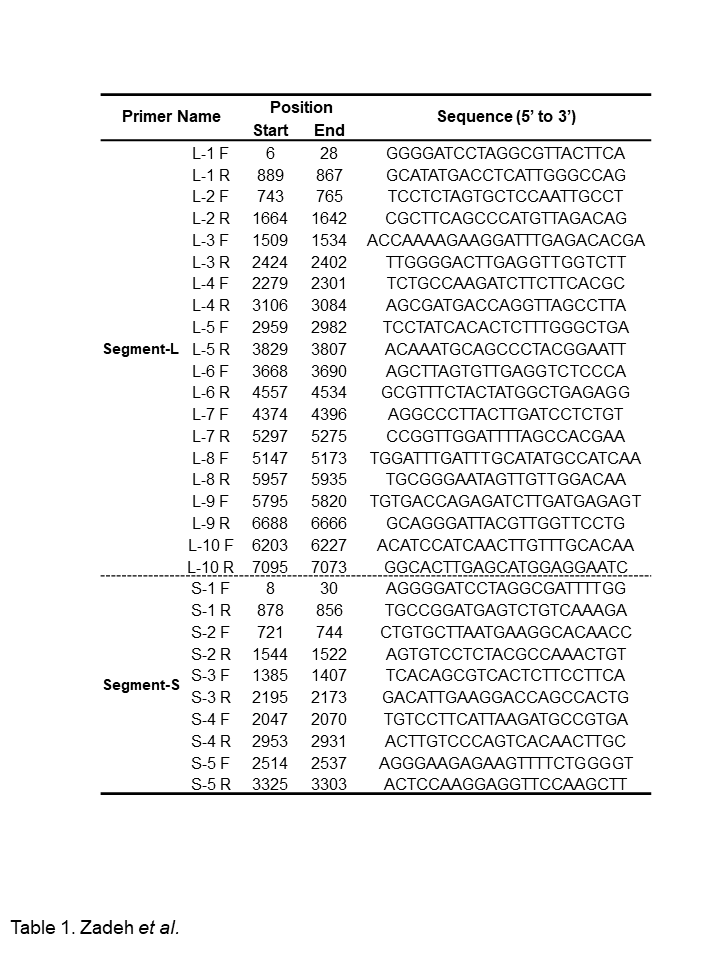

Supplement: S1 Table — (TIF) [file ppat.1010689.s001.tif]

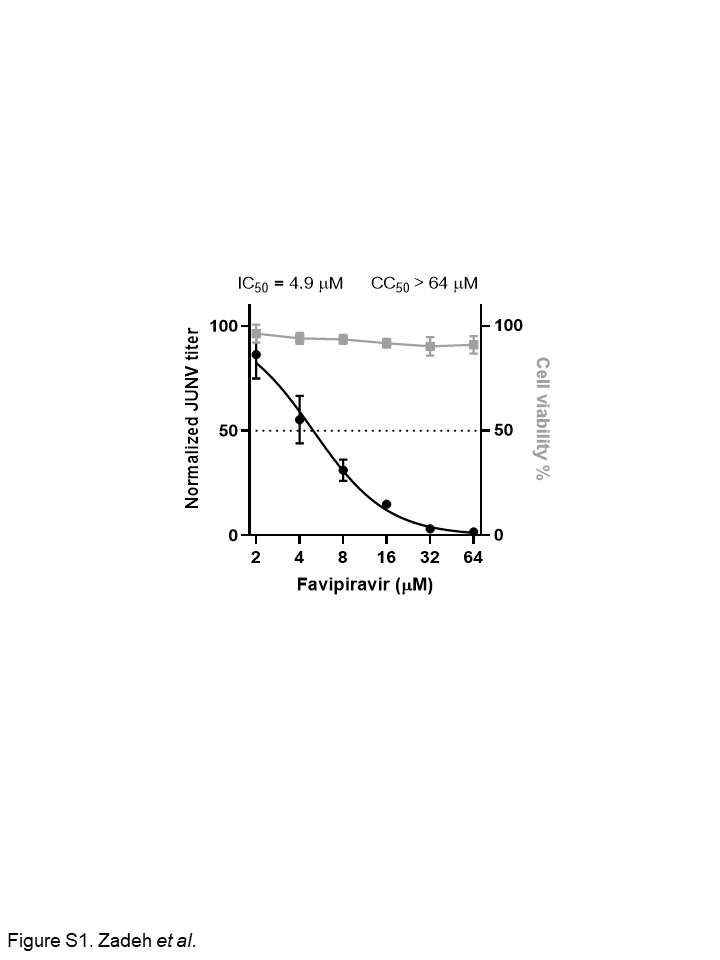

Supplement: S1 Fig — 293T cells were infected with Candid #1 (MOI: 0.1). After adsorption, media containing serial dilutions of favipiravir was added. At 48 hpi, supernatant was collected, and viral titers were determined by plaque assay. Error bars indicate ±SD; three independent experiments in duplicates (n = 6) were performed; nonlinear regression analysis was applied. (TIF) [file ppat.1010689.s002.TIF]

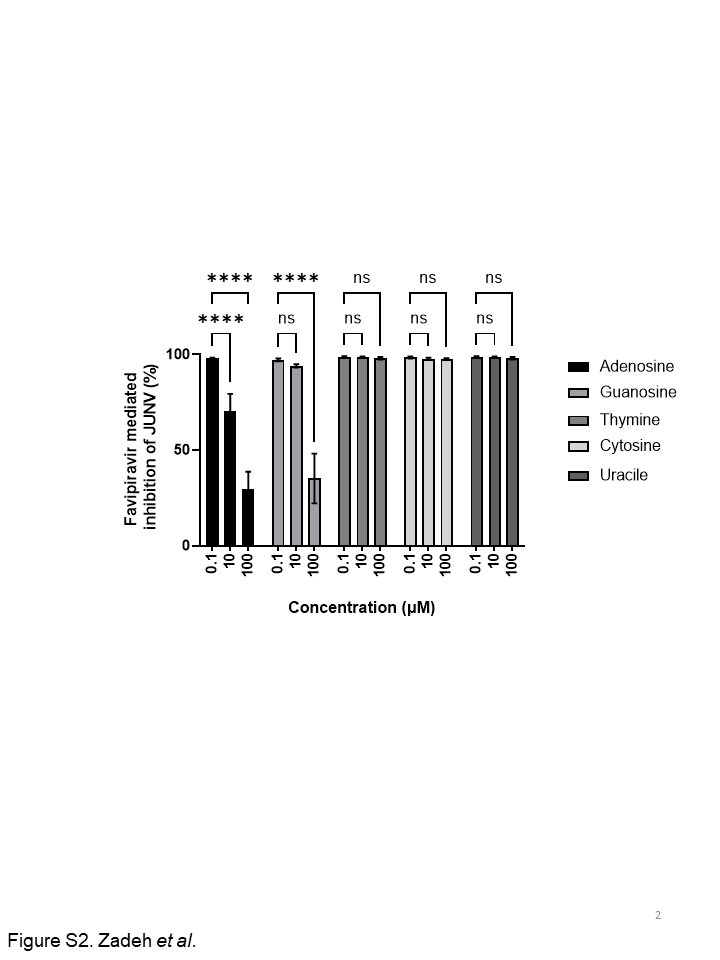

Supplement: S2 Fig — 293T cells infected with JUNV (MOI: 0.01) were treated with serial dilutions of nucleosides adenosine, guanosine, thymine, cytosine, and uracil in combination with 50 μM of favipiravir. At 48 hpi, viral titers were measured by plaque assay. Titers were normalized to anti-JUNV activity of favipiravir to estimate the reversal imposed by nucleotide supplementations. Error bars indicate ±SD; two independent experiments in three biological replicates (n = 6) were performed. Statistical significance was determined by 2-way ANOVA tests (ns indicates not significant and *** indicates P < 0.001). (TIF) [file ppat.1010689.s003.TIF]

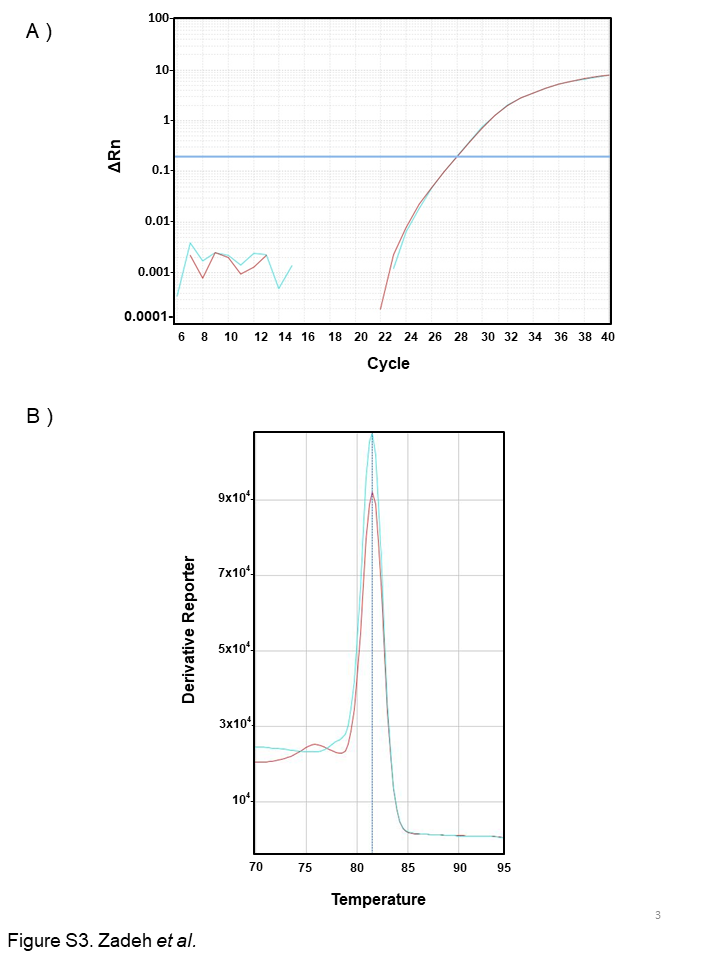

Supplement: S3 Fig — (A) The amplification plot for VSV-M detection from Candid #1pv-A168 (red) or Candid #1pv-T168 (blue) showing a CT value of 27.99 and 27.95 are shown, respectively. (B) Melting curve analysis at the end of the amplification run confirms the specificity of the assay. (TIF) [file ppat.1010689.s004.TIF]

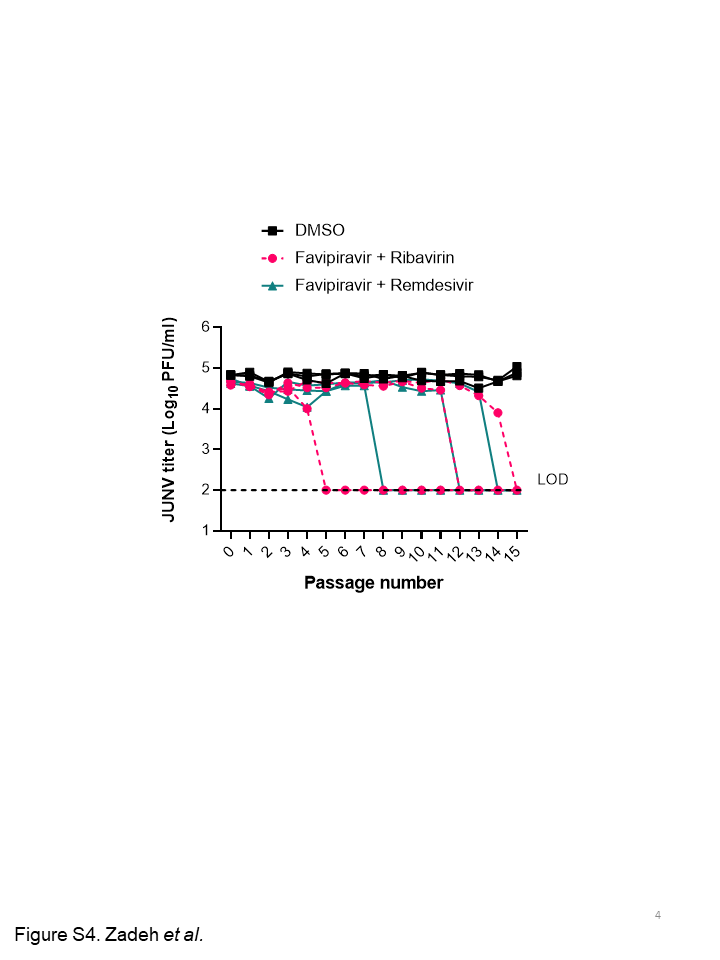

Supplement: S4 Fig — 293T cells were infected with JUNV at MOI of 0.01 for initial inoculation and 10-fold dilutions for the remaining passages (n = 3). After adsorption, cells were treated with combinations of favipiravir (0.3 μM-16.33 times lower than its IC50 value), ribavirin (0.3 μM-20.6 times lower than its IC50 value), and remdesivir (1 nM-240 times lower than its IC50 value). These concentrations were decided based on synergy assay data. Virus was then titrated at 48 hpi by plaque assay. (TIF) [file ppat.1010689.s005.TIF]

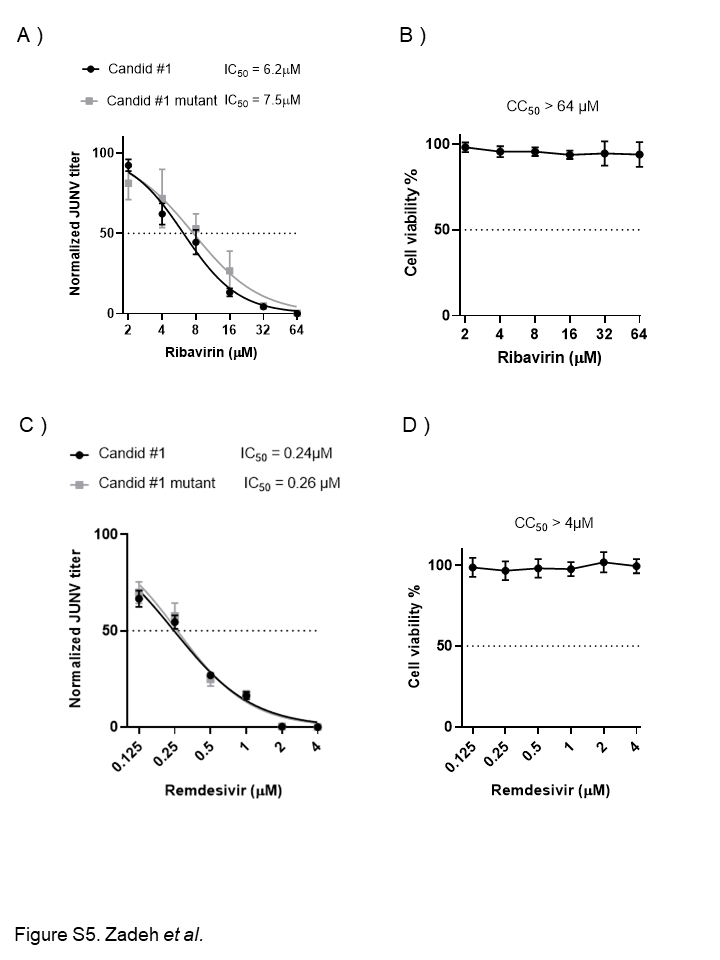

Supplement: S5 Fig — 293T cells were infected with JUNV Candid #1 or Candid #1-mutant virus (MOI: 0.1). Media containing the indicated concentrations of ribavirin was added. At 48 hpi, viral titers were measured by plaque assay. Cytotoxicity assay was performed as described in materials and methods. Error bars indicate ±SD; three independent experiments in duplicate (n = 6) were performed; nonlinear regression analysis was applied. (TIF) [file ppat.1010689.s006.TIF]
