# Supplementary material for: Potential and action mechanism of favipiravir as an antiviral against Junin virus
Source: PLoS Pathog. 2022 Jul 11;18(7):e1010689. doi: 10.1371/journal.ppat.1010689 (PMC9302769; doi:10.1371/journal.ppat.1010689)
Supplement: Attachment — Submitted filename: Response letter_Vahid et al-final.docx [file ppat.1010689.s007.docx]

**ID: PPATHOGENS-D-21-02584 - [EMID:c38c76aeb41d58f6]**

**Article title:** Potential and action mechanism of favipiravir as an antiviral against Junin virus

**Authors:** Vahid Rajabali Zadeh, Tosin Oladipo Afowowe, Haruka Abe, Shuzo Urata, and Jiro Yasuda

Dear Editors and Reviewers,

We would like to express our gratitude to the editors and the reviewers for considering our submission and for their constructive comments on this research work. We added several data and have revised the manuscript accordingly as described below.

Part I – Summary

**Reviewer #1**

**The manuscript covers ground that has been previously examined by a number of groups, many of which are referenced. Previous studies have shown that favipiravir acts as a purine analog (antagonized by authentic purine bases) that introduces mutations and, among other modes of action, pushes the virus to lethal mutagenesis. While the authors conclude that favipiravir resistance can be mediated through increased RdRP fidelity, the evidence for such, and the effect of the N462D mutation itself, is weak (see below). Additionally, others have reported the favipiravir will synergize with ribavirin, another purine analog whose mechanism of action is also complex. Prior studies have shown that ribavirin induces transition mutations, and the authors show the same for favipiravir. Indeed, transitions represent predominant mutations in various RNA viruses, even in the absence of base analogs.**

RE: Thank you for your comments and suggestions to improve this study. We agree that favipiravir has been extensively studied for a number of different viruses including arenaviruses. However, we believe that the biosafety complexities associated with highly pathogenic viruses have hindered investigations on isolation of drug resistant variants of hemorrhagic fever viruses. Junin virus is the only BSL-4 pathogen that has a live-attenuated vaccine strain-Candid #1 (BSL-2) available for clinical use, providing an opportunity to generate knowledge/evidence on drug-resistance in a hemorrhagic fever virus. We have modified the text to clarify our rational and to state what distinguishes our investigation and how that contributes to the existing knowledge in the field.

**The authors description of the A168T mutation in the virus glycoprotein that arises in favipiravir-resistant virus suggests that they are unaware of the significant literature on this mutation. T168A is one of the important attenuating mutations that arose upon selection of the attenuated Candid#1 vaccine strain of JUNV. The mutation removes a glycosylation site and extensive studies from the Paessler lab suggest that the lack of glycosylation results in GPC instability, reduced GPC levels and induction of the unfolded protein response. The Nunberg lab has shown that this mutation in Candid#1 readily reverts back to 168T upon passage in cell culture as the virus is stressed, either by culture conditions or by unrelated mutation (or by favipiravir?). It is possible that by making the virus more fit, the A168T reversion event can contribute to overcoming the barrier imposed by favipiravir. As the effective load of the more-fit virus is greater, more virions will survive an antiviral effect that renders 90% of the virus population noninfectious. An analogous situation – where fitness is enhanced in a manner that overcomes the initial (albeit unrelated) defect - is described by Chen et al PPath 17(12) e1010191. The authors’ reported decrease in specific infectivity in the resistant virus may thus be the result of the N462D mutation in RdRP, as A168T is expected and indeed reported by the authors to increase infectivity (Fig 6).**

RE: Thank you for highlighting that. With a lot of interest, we have followed Paessler and other leading arenavirus laboratories throughout our past and current research with arenaviruses.

In hindsight, we recognize that a fair description of previous research on GPC-168 residue was overlooked as we were going through different stages of manuscript preparation. We would like to state that, to the best of our knowledge, this is the first mechanistic evidence on the importance of T168 residue in virus entry kinetics (as opposed to virus assembly in other reports) that adds to the existing knowledge on Candid #1 attenuation mechanisms. We have made changes in the new version of the manuscript to cover all aspects on functional importance of T168 residue (lines 334-338).

We agree that a more-fit virus with an adapted life cycle can better survive antiviral effect of favipiravir, as we had stated in our discussion. We now modified the text to bring more clarity and accommodate more details (please see Part II – Major Issues: Key Experiments Required for Acceptance, for details).

**Although increased fidelity has been shown to mitigate the effects of base analogs in other systems, the authors’ evidence in this case is weak. The reported reduction in base substitutions in the resistant mutant vs wild-type in the presence of favipiravir is not obviously greater than that reported in the presence of DMSO (21 and 5 versus 5 and 2, respectively), although the absolute numbers are greater in the wild-type than in the mutant (21 and 5, and 5 and 2). Firstly, these numbers are small and conclusions would be strengthened by using global RNA-Seq analysis rather than Sanger sequencing of some 50 individual 450-base clones of NP. (If NP were chosen for sequencing because it does not appear to be a target for favipiravir resistance and thus represents an evolutionarily neutral region, the authors might state their reasoning). More importantly, the reduction in base substitutions may reflect the inhibitory effect of favipiravir treatment on virus replication (greater effect in the wild-type than the resistant mutant, and of course absent in control cultures) and the reduced specific infectivity of the mutant, which would combine to diminish the amount of infectious progeny at each round of virus replication and thereby the amount of RNA replication and the opportunity for mutation. Additional studies are needed to clarify the significance of both mutations. It would be interesting to see if N462D arises upon passage of recombinant A168T Candid#1, or if the A168T virus is itself less sensitive to favipiravir. If the mutation were to increase fidelity, then we might expect to see stronger antagonism by authentic purine bases (see Fig S2) but this study was not performed. If RdRP fidelity is increased, it does not appear to extend to ribavirin or remdesivir as the favipiravir-resistant virus remains sensitive to these (Fig. S5), suggesting that N462D, if indeed responsible for enhanced fidelity, may be specific to favipiravir. Taken together, it is difficult to assert that RdRP fidelity is increased or that N462D is responsible for this or favipiravir resistance.**

RE: Our conclusion with respect to higher fidelity phenotype of P11 comes from two independent bodies of evidence. First, following an established approach that has been shown to be sensitive enough to distinguish fidelity differences by targeting regions as few as 100bp ([Beaucourt](https://www.jove.com/author/St%C3%A9phanie_Beaucourt) et al., 2011), we determined mutation frequencies within NP gene (450 bps). We chose NP because it is a region prone to mutation (Ana Grande-Pérez et al. 2015), and therefore, we could see a baseline in our non-treated controls. We added the description on this in the manuscript (lines 186-189). We agree that a global sequence analysis would show the difference better and that is possibly the reason we could not detect a significant difference between the two viruses in absence of favipiravir, but the goal of the experiment was to show how in presence of favipiravir both polymerases would behave. Secondly, as the reduction in specific infectivity is the hallmark of error-catastrophe (Arias et al., 2014), we reason that lower specific infectivity of Candid #1-resistant virus is the second piece of evidence that supports our conclusions (please see below for further details). We also agree with the fact that stronger antagonism would be expected with purine base if resistant virus was used. But the sensitivity of the assay is insufficient to detect it (high concentration of favipiravir and purines should be added in order for us to be able to observe a meaningful difference which won’t allow detection of synergistic effect).

**Reviewer #2**

**In “Potential and action mechanism of favipiravir as an antiviral against Junin virus” Zadeh et al. describe the generation of favipiravir-resistant JUNV mutants, and further investigate the potential mechanisms of resistance. The objectives of the study are well defined, with a strong experimental method, clear figures, and well written results and discussion sections. The data presented is sound, and represents a thorough investigation into the resistance mechanisms conferred by the two mutations identified.

The main comments I have on the data that would strengthen the manuscript are that: 1) relative appearance of the two identified mutations; 2) some data that has been generated is not included in this manuscript (data not shown); and 3) no data investigating these mutations in the more clinically relevant virulent JUNV strains.**

RE: Thank you for the critical evaluation of our study. According to your comment, 1) we performed a Next Generation Sequencing to trace the emergence of both variants during passage study (Figure 2B and please see Part II – Major Issues: Key Experiments Required for Acceptance for further details). We believe that it has strengthened our conclusions. 2) We also added the data which wasn’t shown previously (Figure 6B). 3) Unfortunately, there is no BSL-4 facility available for us to do the experiment using the more clinically relevant virulent JUNV strains. In addition, if we are available to use a BSL-4 facility, the generation of drug-resistant mutants of BSL-4 viruses which are not attenuated should be considered carefully. Therefore, we cannot respond to this request at present, although it may be tried in future.

Part II – Major Issues: Key Experiments Required for Acceptance

**Reviewer #1**

**The authors description of the A168T mutation in the virus glycoprotein that arises in favipiravir-resistant virus suggests that they are unaware of the significant literature on this mutation. T168A is one of the important attenuating mutations that arose upon selection of the attenuated Candid#1 vaccine strain of JUNV. The mutation removes a glycosylation site and extensive studies from the Paessler lab suggest that the lack of glycosylation results in GPC instability, reduced GPC levels and induction of the unfolded protein response. The Nunberg lab has shown that this mutation in Candid#1 readily reverts back to 168T upon passage in cell culture as the virus is stressed, either by culture conditions or by unrelated mutation (or by favipiravir?). It is possible that by making the virus more fit, the A168T reversion event can contribute to overcoming the barrier imposed by favipiravir. As the effective load of the more-fit virus is greater, more virions will survive an antiviral effect that renders 90% of the virus population noninfectious. An analogous situation – where fitness is enhanced in a manner that overcomes the initial (albeit unrelated) defect - is described by Chen et al PPath 17(12) e1010191. The authors’ reported decrease in specific infectivity in the resistant virus may thus be the result of the N462D mutation in RdRP, as A168T is expected and indeed reported by the authors to increase infectivity (Fig 6).**

RE: We regret that we failed to have a fair description of previous studies on this mutation, and we have modified the manuscript to accommodate them. In addition, we discussed what our findings added to the existing knowledge (lines 333-337). As we described (lines 342-345) in the discussion, we agree with you that a more-fit variant has a higher chance of survival under the pressure of favipiravir, thus require a higher IC_50_ value. Thank you for your valuable comment.

**Although increased fidelity has been shown to mitigate the effects of base analogs in other systems, the authors’ evidence in this case is weak. The reported reduction in base substitutions in the resistant mutant vs wild-type in the presence of favipiravir is not obviously greater than that reported in the presence of DMSO (21 and 5 versus 5 and 2, respectively), although the absolute numbers are greater in the wild-type than in the mutant (21 and 5, and 5 and 2). Firstly, these numbers are small and conclusions would be strengthened by using global RNA-Seq analysis rather than Sanger sequencing of some 50 individual 450-base clones of NP. (If NP were chosen for sequencing because it does not appear to be a target for favipiravir resistance and thus represents an evolutionarily neutral region, the authors might state their reasoning). More importantly, the reduction in base substitutions may reflect the inhibitory effect of favipiravir treatment on virus replication (greater effect in the wild-type than the resistant mutant, and of course absent in control cultures) and the reduced specific infectivity of the mutant, which would combine to diminish the amount of infectious progeny at each round of virus replication and thereby the amount of RNA replication and the opportunity for mutation. Additional studies are needed to clarify the significance of both mutations. It would be interesting to see if N462D arises upon passage of recombinant A168T Candid#1, or if the A168T virus is itself less sensitive to favipiravir. If the mutation were to increase fidelity, then we might expect to see stronger antagonism by authentic purine bases (see Fig S2) but this study was not performed. If RdRP fidelity is increased, it does not appear to extend to ribavirin or remdesivir as the favipiravir-resistant virus remains sensitive to these (Fig. S5), suggesting that N462D, if indeed responsible for enhanced fidelity, may be specific to favipiravir. Taken together, it is difficult to assert that RdRP fidelity is increased or that N462D is responsible for this or favipiravir resistance.**

RE: As stated above, we provided the experimental evidence from different methodologies that reassure higher fidelity of resistant variant and based on that evidence, we made our conclusions. To clarify the precise role of each mutation, we performed a next generation sequencing analysis and could show the co-relation between the emergence of a mutation and the acquisition of resistance to favipiravir (Figure 2B). Both mutations gradually increased and constituted the maximum frequency at P10 (just before isolation of resistant virus) making it hard to speculate the role of GP168. We agree with your comment and the only hypothesis that we currently have is the fitness theory. We consider it an interesting research question that is worth a follow up research in combination with animal studies.

**Reviewer #2**

**Firstly, in figure 1 it is apparent that between P10 and p11 the resistant mutation (with the 2 mutations) was generated. However, the authors only present sequence data from the P0 and P11 strains. I think the manuscript would be strengthen if the viruses at some of the intermediate passage numbers were also sequenced, and therefore the relative appearance of each individual mutation could be elucidated. From a readers point of view, I would be interested in knowing (especially given the later data examining the relative importance of each mutation) whether one came before the other, or did both appear at P11 together? This would also generate data which could strengthen the conclusions on the discussion, knowing which of the mutations (if any) is the more relevant for the resistance observed here.**

RE: Thank you for your valuable suggestion. According to your suggestion, we performed NGS analysis to determine the relative population of mutants in different passages and we observed gradual accumulation of both mutations under the influence of favipiravir. Interestingly, GPC-A168T population emerged earlier in higher proportion as compared to RdRp-N462D substitution. The complete replacement of RdRp-N462D in P11 corelated with the acquisition of drug-resistance, further supporting our conclusion. We included these results in the revised version of this manuscript (Figure 2B).

**Secondly, one comment I had regarding the GPC-A168T mutation was regarding the effect this had on cellular entry, specifically attachment. Although the experiment was performed (with the pseudotype) these data were not included in the manuscript. I think however that, if possible, these data should be included as a panel in figure 6 as I believe, even if it is negative data, it is an important finding. It would also clearly demonstrate the thoroughness to which the authors have investigated the potential mechanisms of the GPC-A168T mutation.**

RE: Thank you for your important suggestion. As you requested, we added the data to Figure 6 (Figure 6B). The result showed that there was no significant (t test) difference in virus attachment efficiencies (lines 244-247).

**Finally, although the authors state that there is no reverse genetics system for JUNV, there is published data of a system (Albariño et al. Efficient reverse genetics generation of infectious Junin viruses differing in glycoprotein processing. J Virol. 2009 PMID: 19321606). However, I understand that without a BSL4 lab this would be impossible to set up, I wondered if the authors had given any thought to collaborating with a lab that does have this facility to generate a JUNV with these mutations to look at resistance in more clinically relevant strains?**

RE: Thank you for highlighting it. As you inferred, we meant to state “in absence of reverse genetics system in our facility”. We corrected the text to clarify it (line 351). We agree with your comment. It may be considered when our BSL-4 facility will be ready to use.

Part III – Minor Issues: Editorial and Data Presentation Modifications

**Reviewer #1:**

1. **line 58-59: might re-order to read “...disease that is endemic in Argentina and caused by...”**

RE: According to your suggestion, we modified this sentence (lines 58-59).

2. **line 61: rather than the “few reports” on the use of immune plasma therapy, the authors should know that this therapy is used routinely in Argentina.**

RE: We corrected the sentence (line 62).

3. **line 83: might replace “antiviral” with the more specific “favipiravir”, and “high” with “strong”.**

RE: We replaced “Antiviral” with “favipiravir” (line 84).

**4. line 93: might replace “invalidates” with “abolishes”.**

RE: The word “invalidates” was changed to “abolishes” (line 94).

**5. line 107-109: please clarify – as Candid#1 vaccination is quite effective, why would one need to consider favipiravir therapy?**

RE: We deleted this description.

**6. para starting line 112: might introduce and provide background information on Candid#1.**
RE: According to your suggestion, at the beginning of this paragraph, we described the background information on Candid#1 (lines 114-119).

**7. line 126: this is the first and only mention of “defective interfering particles”, a term that has specific meaning in virology (and in some arenavirus work). If the authors mean to refer to non-infectious genomes containing favipiravir-induced mutations, they might stick with the this nomenclature to avoid confusion with DIPs. Nowhere is “interference” with infection by noninfectious particles discussed.**
RE: Thank you for your comment. We revised this sentence (lines 129-132).

**8. line 146: by “same mutations...as P0 parental virus”, are the authors referring to Candid#1-specific changes relative to pathogenic JUNV (including A168)? One wouldn’t expect too many other changes in a P0 stock. Please clarify.**

RE: We apologize for the confusion. We meant that the sequencing result from every clone was identical to our initial sequencing of supernatant prior to clonal isolations. We think this information was not relevant. Therefore, we deleted this sentence.

**9. Fig 3: it is remarkable that the authors find extensive virus production at 8 hr post-infection (~3.5 log10 vs ~4.1 log10 at 24 hr). This is a considerably shorter eclipse period than commonly reported by others.**
RE: To the best of our knowledge, 293T cells have not been used in the studies to see JUNV production so far. It may be the reason why the virus showed rapidly growth.

**10. line 179: please clarify or cite how the “tolerable threshold of error catastrophe” is “estimated”.**
RE: The estimation comes from numerous research work on RNA viruses. We modified the text to explain that (lines 196-198).

**11. line 192: the error bias is observed in the virus population and not the “transitional mutations”. Do the authors refer to “transition” substitutions, mutation that arise transiently during passage, or mutants (vs mutations) that arise on passage? Please clarify.**

RE: We corrected the text (line 210).

**12. line 208-210: It is likely that multiple rounds of infection take place in 48 hr after a low moi infection, and thus it likely that the lack of difference in final RNA copy number reflects more the duration of each cycle (allowing maximal spread of the more sensitive virus to achieve the same concentration as the less sensitive one) than to any differential inhibition. Please clarify how RNA copy number is the same despite differential sensitivity to favipiravir.**

RE: While we agree to your comment that we cannot exclude the possibility of multiple rounds of infection, which may affect our observations, it has been shown that presence of a mutagen can lead to production of non-infectious viral particles (Ana Grande-Pérez, et al., PNAS, 2005) yet impairs virus infectivity. That can be still detected with PCR. We modified the text to clarify it (lines 228-230).

**13. line 223, Fig 6A: it is unclear how much weight to place on the one point showing a difference (16 hr) when the two other points (8 and 24 hr) show no difference. Thus, the notion that entry is more rapid in the 168T pseudovirus is questionable.**

RE: We repeated the assay with an additional time point and see a difference at 12 hpi as well (New Figure 6A). Inherent limitations of the assay do not allow further expansion of time points as the signal begins to plateau. And it is the reason why we also used a different detection method (Figure 6C and D) to strengthen our conclusion.

**14. line 243: might specify that “no...effect” is in the absence of favipiravir.**
RE: “In the absence of favipiravir” was added (line 267).

**15. line 249-251 (and line 291): please explain how Fig 7F speaks to chain termination?**
RE: As shown in Fig 7, low concentrations of favipiravir did not affect nluc mRNA levels as detected by qPCR and also did not affect minigenome signal up to 200 µM. Above that concentration, mRNA levels began to fall, suggesting pre-mature mRNA synthesis (chain termination) and the minigenome signal also began to reduce. It unfortunate that minigenome assay is not sensitive to mutagenesis event (25 to 200 µM). We otherwise would be able to show higher fidelity of RdRp-D462 using this system. We have further explained our reasoning to clarify it (lines 273-276).

**16. line 267-269: might reword that it’s the gradual increase in selective pressure (favipiravir concentration; not “mutant viruses”) that enables adequate levels of replication to support mutation and the emergence of resistant viruses. This is a typical design for selection.**
RE: We rewrote the sentence (lines 292-293).

**17. line 293: the authors claim ribavirin is non-mutagenic, yet literature claims otherwise? Please clarify.**

RE: Ribavirin is known to act both by mutagenic and non-mutagenic mechanisms (lines 319-321). We modified the text to clarify our statement.

**18. line 321-323, and Fig S4: the failure to identify resistance to the combination of favipiravir and ribavirin/remdesivir is rather unsurprising – at a minimum, one would need 2 (vs 1) mutation, and it is unclear whether the effective (synergistic) potency was considered in establishing selection conditions – selection may fail if replication is excessively reduced. However, Fig S4 appears to show no antiviral effect of the combination, as DMSO and combination tracks overlap (compare with Fig1A, where treatment with favipiravir alone does reduce virus output). This too would explain the absence of selection. Please explain.**

RE: Drug concentrations were decided based on our synergy assay experiment (Figure 8). As you accurately mentioned, excessive inhibition would not allow for selection. Thus, we wanted to have minimum inhibitory effect of the drug. This explains why we do not see any reduction in viral titers for first few passages (the reduction might be below the detection sensitivity of plaque assay) and as we passage the virus the cumulative effect is observed. We have modified figure legend to explain it (lines 918-921).

**Reviewer #2:**

**Figure 1: I think it would be helpful to indicate on the x-axis where the dose of T705 was increased, or indicate the dose at each passage maybe?**
RE: Thank you for your suggestion. We indicated it in Figure 1.

**Figure 6A: I think y-axis should say normalized? And indicate % as a unit?**
RE: Thank you for your comment. We corrected the labels in Figure 6A.

**Line 344: Please list Genbank number of Cd#1 strain used (if available)**
RE: Unfortunately, we do not have a GenBank number associated with this strain. Instead, we added the reference study from that we obtained our stock. We hope that it would help the readers (line 381).

**Line 733: “LOD” not shown on graph but listed in legend.**
RE: We removed it from the legend.

**Line 741: Should say “cells” and not “cell”.**
RE: We corrected it (line 832).

**Line 747: LOD is shown on graph but not explained in legend.**
RE: We added the explanation to LOD (line 838).

**Line 761: Unsure how the n is 8 for this?**
RE: Thank you for the corrections. We corrected it. Replicates are now described accurately (lines 851-852).

**Line 815: Unsure how the n is 6 for this?**
RE: Thank you for the corrections. We corrected it. Replicates are now described accurately (line 880).

**Lie 831: Should say “cells” and not “cell”.**

RE: We corrected it (line 925).

We hope that we have addressed all concerns raised by the reviewers in this revised version of the manuscript, and that this work would be reconsidered for publication in PLOS pathogens.

Sincerely yours,

Jiro Yasuda
